# Structural Insights into Protein-Protein Interactions Involved in Bacterial Cell Wall Biogenesis

**DOI:** 10.3390/antibiotics5020014

**Published:** 2016-04-28

**Authors:** Federica Laddomada, Mayara M. Miyachiro, Andréa Dessen

**Affiliations:** 1 Institut de Biologie Structurale (IBS), University Grenoble Alpes, Grenoble F-38044, France; federica.laddomada@ibs.fr; 2 Centre National de la Recherche Scientifique (CNRS), IBS, Grenoble F-38044, France; 3 Commissariat à l’Energie Atomique et aux Energies Alternatives (CEA), IBS, Grenoble F-38044, France; 4 Brazilian National Laboratory for Biosciences (LNBio), CNPEM, Campinas, São Paulo 13083-100, Brazil; mayara.miyachiro@lnbio.cnpem.br

**Keywords:** peptidoglycan, elongation, cell division, protein complexes, Mur enzymes, MraY, bacterial cytoskeleton

## Abstract

The bacterial cell wall is essential for survival, and proteins that participate in its biosynthesis have been the targets of antibiotic development efforts for decades. The biosynthesis of its main component, the peptidoglycan, involves the coordinated action of proteins that are involved in multi-member complexes which are essential for cell division (the “divisome”) and/or cell wall elongation (the “elongasome”), in the case of rod-shaped cells. Our knowledge regarding these interactions has greatly benefitted from the visualization of different aspects of the bacterial cell wall and its cytoskeleton by cryoelectron microscopy and tomography, as well as genetic and biochemical screens that have complemented information from high resolution crystal structures of protein complexes involved in divisome or elongasome formation. This review summarizes structural and functional aspects of protein complexes involved in the cytoplasmic and membrane-related steps of peptidoglycan biosynthesis, with a particular focus on protein-protein interactions whereby disruption could lead to the development of novel antibacterial strategies.

## 1. Introduction

Peptidoglycan (PG) is a key component of the bacterial cell wall, and plays an important role in bacterial shape, as well as division and elongation processes. In addition, it serves as anchor for surface-exposed virulence factors, secretion systems, and other cell wall-associated molecules such as teichoic acids and lipopolysaccharide. Its mesh-like structure surrounds the entire bacterial cell and is composed of polymerized GlcNAc and MurNAc moieties whose associated stem peptides are cross-linked [1,2,3]. Disruption of PG architecture or its biosynthesis may lead to cell rupture and death, as illustrated by the action of β-lactam antibiotics that target the last steps of PG biosynthesis [1,4,5,6,7].

**Figure 1:**
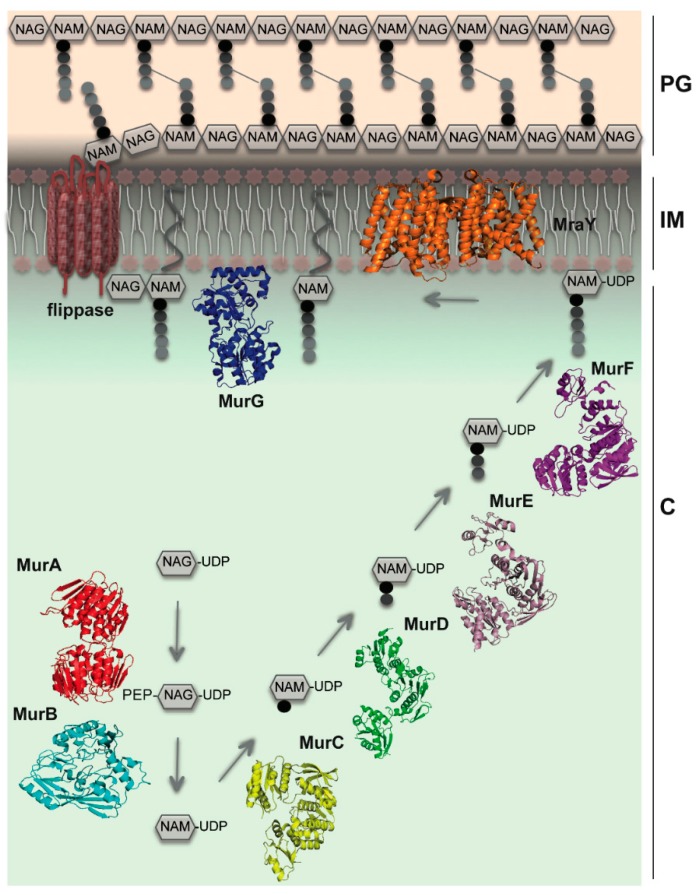
A simplified view of cytoplasmic and membrane-related steps of PG biosynthesis. The concerted action of MurA and MurB generates the initial precursor, UDP-N-acetyl muramic acid (UDP-NAM). Mur ligases (C–F) catalyze the stepwise addition of a pentapeptide to UDP-N-acetyl muramic acid (UDP-NAM). MraY anchors the UDP-NAM-pentapeptide unit to the inner membrane through an undecaprenyl phosphate carrier lipid, forming lipid I. MurG participates in the formation of the final peptidoglycan building block (Lipid II), which is then flipped to the periplasm by flippases. C: cytoplasm; IM: inner membrane; PG: peptidoglycan layer. Cytoskeletal elements are not shown for simplicity. PDB codes of molecules depicted here: MurA (1NAW); MurB (1MBT); MurC (1J6U); MurD (4BUC); MurE (4BUB); MurF (3ZL8); MurG (1F0K); MraY (4J72).

In rod-shaped bacteria, the orchestration of cellular morphogenesis occurs in two phases: cell division, which generates two daughter cells, and elongation, where cellular growth occurs along the longitudinal axis of the cell. Both phases require synthesis, modification, and recycling of PG. The multistep process that leads to the synthesis of PG occurs in three different cellular compartments, involving mostly enzymes that work sequentially (Figure 1). The first stage of PG biosynthesis occurs in the cytoplasm, and involves the synthesis of UDP-N-acetylmuramyl-pentapeptide (UDP-MurNAc-pentapeptide) from UDP­N-acetyl-glucosamine (UDP-GlcNAc) via the action of enzymes MurA through MurF. Cytoplasmic steps also involve two cytoskeletal proteins: the actin homolog MreB, that plays a key role in shape determination, and FtsZ, that forms a contractile ring at the future cell division site [4,6,7]. The second stage, which occurs at the inner face of the cytoplasmic membrane, involves the activity of two essential enzymes, MraY and MurG. The integral membrane protein MraY catalyzes the transfer of the phospho-MurNAc-pentapeptide motif of UDP-MurNAc-pentapeptide to a lipid carrier, undecaprenyl phosphate, to form Lipid I [8,9]. The final cytoplasmic step involves the link between Lipid I and a GlcNAc molecule by the membrane-associated enzyme MurG, generating Lipid II [9,10], which is eventually translocated to the periplasmic side of the cell by flippases [11,12,13,14]. Finally, in the periplasm, Penicillin-Binding Proteins (PBPs) incorporate the GlcNAc-MurNAc-pentapeptide into the PG layer through glycosylation and transpeptidation reactions [1,15]. Several of the proteins involved in the aforementioned processes have been suggested as being members of distinct protein complexes (the “divisome,” in cell division, and the “elongasome,” in cell wall elongation) [16]. These multi-protein assemblies involve elements of the bacterial cytoskeleton and those of the peptidoglycan biosynthetic machinery [7,17]. The disruption of key interactions between protein partners could provide a mechanism by which to block bacterial cell growth, leading to the development of novel antimicrobial agents. Protein interactions that have been shown to play key roles within cytoplasmic and membrane-embedded steps are the subject of this review.

## 2. MreB Orchestrates Elongasome Assembly

The actin homolog MreB is essential for shape maintenance and cell wall elongation, and its depletion or inhibition causes loss of shape and eventual cell lysis, also affecting cell polarity and even chromosome segregation in some species [18,19,20]. Depending on the visualization technique used, MreB has been reported to adopt either a helical pattern of elongated filaments that run along the cellular periphery or a patched pattern that performs a circumferential rotation around the long axis of the cell [21,22,23,24,25,26]. The issue of the precise nature of MreB has been the subject of much controversy, and although its discussion is not the objective of this review, it is of interest to note that recent work suggests that MreB forms extended, antiparallel filaments that associate to the inner membrane, and that it is capable of coordinating the activity of multiple peptidoglycan synthases. These observations also indicate that MreB has the potential to promote long-range interactions with other proteins involved in cell wall biosynthesis [27,28,29,30,31] and that it could separately coordinate cytoplasmic and periplasmic PG biosyntesis complexes [32].

Numerous techniques have been used to study the interaction between MreB and proteins that form the elongasome, and the cytosolic protein MurG seems to play a key role. Fluorescence microscopy, co-pelleting assays, two-hybrid analyses, and surface plasmon resonance experiments suggest a direct association between MreB and MurG [33,34,35]. Furthermore, immunofluorescence microscopy experiments using either MurG-mCherry fusions or affinity purified MurG antisera reveal a banded pattern of localization along the length of the cell that is dependent on MreB and can be disrupted by the MreB inhibitor A22 [34,36]. These results suggest that MreB and MurG work in tandem in early PG biosynthetic steps at the cytoplasmic side of the inner membrane, serving as scaffolds for the elongasome [33]. It is of note that immuno-precipitation and bacterial two-hybrid studies suggest that MraY is also an integral member of this complex (see below) [34,35,37].

The detection of MurG as a partner of MreB in a number of bacterial systems evokes the question of the potential participation of other Mur ligases as members of such a complex. The idea of the existence of a major cytoplasmic structure involving MreB, MraY, and most Mur ligases has been suggested as a means to provide an explanation to the fact that despite years of structure-guided small molecule development for MurC-G enzymes, leading to the identification of a number of inhibitors that targeted the enzymes *in vitro*, to date few of these molecules have been shown to successfully block bacterial growth *in vivo*. This result could potentially be explained by the masking of Mur active sites within a multi-membered “enzyme cluster” [34,38]. If such a complex does exist, one should be able to show the participation of a number of cytoplasmic PG biosynthesis enzymes in such an assembly. Initial evidence for this possibility was obtained through co-localization experiments of MurC-F by immunofluorescence using mCherry-tagged versions of Mur enzymes, shown to be present in banded patterns as described for MreB above [34]. In addition, co-pelleting and surface plasmon resonance also showed that individual Mur enzymes recognized MreB, MraY and MurG [33,35]; interestingly, MurF was shown to interact directly with MreB in different species [35,39]. However, MurD, MurE, and MurF were not able to bind to each other in the absence of either MreB or MurG [33,40]. These data provide additional indications for the essential scaffolding role of the two latter proteins in a major cytoplasmic complex. The precise characterization of the surface regions employed by MurG (or MreB) to interact with other Mur proteins should provide key information regarding areas that could be targeted by small molecule inhibitors. In these cases, the goal of such inhibition would not be the direct interruption of the stem peptide formation process, but destabilization of the cytoplasmic Mur cluster, leading to a potential block in Lipid I biosynthesis.

## 3. MraY as a Central Player

MraY is a 10-TM, integral membrane protein that associates phospho-MurNAc-pentapeptide, the product of the reaction catalyzed by MurF, to the lipid carrier undecaprenyl phosphate (C55-P), in a step that generates Lipid I and precedes the action of MurG (Figure 1). This essential reaction is one of the few that involves a cytoplasmic PG biosynthesis substrate and which is successfully inhibited by natural antibiotics, being the target of five classes of natural product inhibitors, including tunicamycin, mureidomycin A and liposidomycin B [41]. In addition, MraY is also naturally inhibited by the bacteriolytic E peptide of bacteriophage fX174, which does not target MraY’s active site but rather inserts between the TM domains, thus preventing a key association with other membrane-embedded proteins [42,43]. Recently, several natural product analogs have displayed promising activity against a number of Gram-positive agents, including MRSA [44], while combined MraY/MurG screens have yielded inhibitors of Gram-negative MraY in the micromolar range [45]. In addition, further MraY-aimed drug development, which could target either its catalytic activity or its partner interaction regions, should profit immensely from the reporting of its recent crystal structure [46].

**Figure 2:**
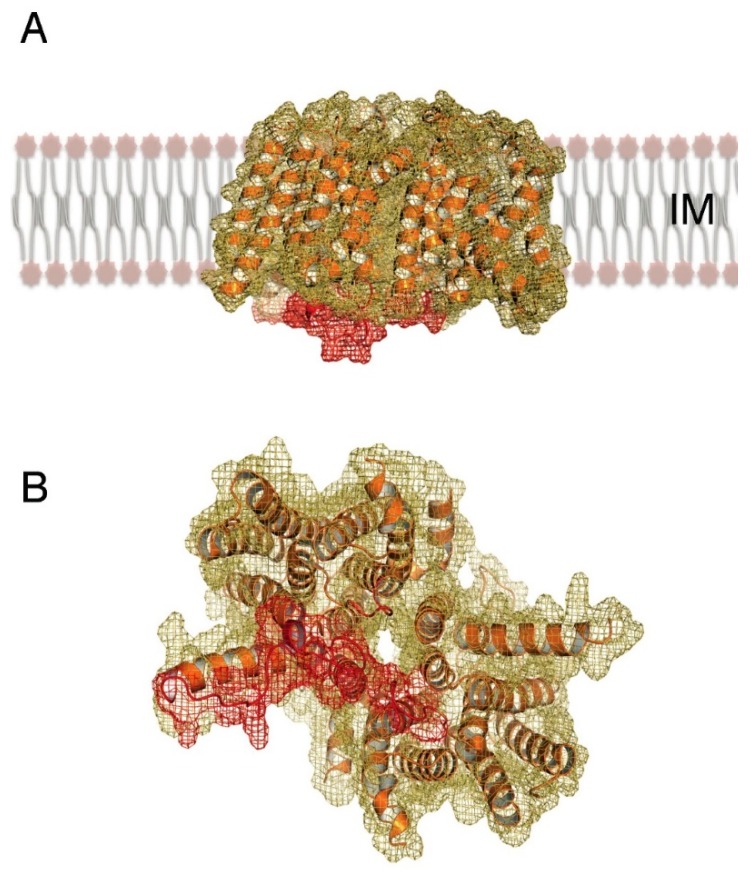
The crystal structure of MraY from *Aquifex aeolicus* (PDB 4J72) reveals (**A**) a dimer displaying 10 TM helices per monomer, whose N- and C-termini face the periplasm. (**B**) A view from the cytoplasmic side indicates a tunnel formed in the monomer-monomer interaction region, buttressed by cytoplasmic loops (in red) that could interact with substrate, partner proteins, or both.

MraY is a dimeric molecule whose active site cleft, as well as a number of loops suggested to play roles in sugar recognition, are located within the inner leaflet of the membrane and face the cytoplasm (Figure 2). This region could also potentially participate in the recognition of partners such as MurF and MurG, as indicated above [34]. It is of interest that Chung and co-workers could not visualize loop A, that connects TMs 1 and 2 [46], which leads to the hypothesis that this loop could require recognition by a cytoplasmic partner for stabilization. These observations suggest that a membrane-associated multi-partite complex involving MurF, MraY, and MurG could catalyze the metabolism of the substrate of MurF (UDP-NAM-tripeptide) all the way through to formation of bilayer-associated Lipid II, the product of MurG, diminishing diffusion of reaction intermediates into the cytoplasm. The characterization of MurG and MreB as scaffolds for other Mur ligases, including MurC, MurD, MurE and MurF strongly suggests that the existence of a complex of higher order than a tripartite form is possible, bringing forth the theory of ”metabolic channeling” in cytoplasmic reactions of PG biosynthesis [35,38]. Notably, this complex could also include MreD, RodA and FtsW, membrane-embedded proteins shown to play roles in partner association, regulation of Mur localization within the cell cycle, and Lipid II flipping to the periplasm [34,40].

## 4. Channeling of PG Building Blocks

**Figure 3:**
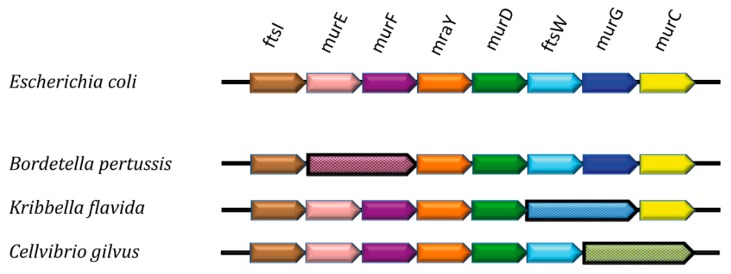
Schematic representation of a section of the *dcw (division and cell wall)* cluster in different bacterial genomes. Adjacent arrows represent contiguous genes involved in cell wall synthesis and division. In a number of species, adjacent genes are fused, such as in numerous strains of *B. pertussis* (murE/murF), and in the actinomycetes *K. flavida* (ftsW/murG) and *C. gilvus* (murG/murC).

Support for the theory of metabolic channeling in PG biosynthesis can also be inferred from genomic analyses of the *mur* cluster. By searching for homologs of MurE in genomic sequences of Gram-negative and Gram-positive species, we identified that a number of proteobacteria as well as bacteroidetes lack individual MurE and MurF variants. Instead, they carry interconnected MurE-MurF forms, where the individual ligase-encoding genes are fused by a linker which corresponds to approximately 20 residues (Figure 3).

The potential advantage of expressing in tandem Mur ligases that catalyze sequential reactions is evident, and points to the possibility that the catalytic sites of these enzymes could be arranged to channel the product of the MurE reaction directly into the MurF active site cleft. Interestingly, we were also able to identify fusions between MurG and MurC-encoding genes, which do not catalyze sequential reactions; however, these observations point to a model where a multi-enzyme PG biosynthesis complex could exist in the cytoplasm, at least during specific points of the cell cycle. Recently, an enzyme of the branching diamino-pimelate pathway was also shown to interact with MurE and MreB [32], an observation which further supports the existence of a network of key interactions within the cytoplasm. More insight into this exciting possibility will require structural information on Mur ligase and PG biosynthetic complexes.

## 5. Linking MreB’s Cytoplasmic and Periplasmic Functionalities

**Figure 4:**
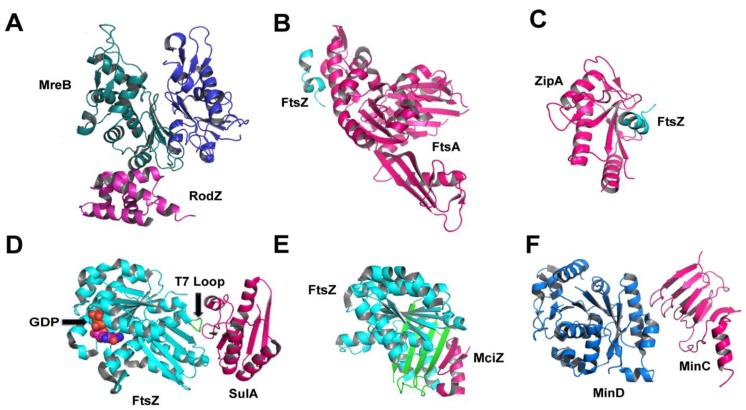
Protein interactions involving cytoskeletal proteins that play key roles in cytoplasmic and membrane-embedded PG biosynthesis steps. (**A**) MreB:RodZ from *T. maritima* (2WUS), where MreB’s subdomains IA and IIA are shown in blue and green, respectively; (**B**) FtsA:FtsZ (res 338–351) from *T. maritima* (4A2A); (**C**) ZipA:FtsZ (res 367–383) from *E. coli* (1F47); (**D**) SulA:FtsZ from *P. aeruginosa* (1OFU); (**E**) MciZ:FtsZ from *B. subtilis* (4U39), where MciZ‘s β-hairpin completes FtsZ’s 4-stranded sheet ; (**F**) MinC:MinD from *A. aeolicus* (4V02).

The filaments or patches formed by MreB have been suggested as forming “tracks” for PG synthases and hydrolases that act within the periplasmic space. The question of how MreB “links” cytoplasmic and periplasmic PG biosynthesis partners was answered by the discovery of the cytoskeletal protein RodZ, an inner membrane protein containing an 80-residue, N-terminal cytoplasmic region and a 200-amino acid periplasmic C-terminal tail [47,48,49]. RodZ is required for cell shape maintenance, and co-pelletting assays as well as calorimetry and light microscopy experiments indicated that the cytoplasmic domain of RodZ binds both the monomeric and polymerized forms of MreB [50]. MreB folds into two major domains (I and II), each being subdivided into two further subdomains (IA, IB, IIA, IIB; [51]). The crystal structure of the complex between MreB and RodZ’s cytoplasmic region (Figure 4) reveals that RodZ interacts directly with subdomain IIA and is sandwiched between two MreB monomers. Despite this fact, binding occurs in such a way that is compatible with both a monomeric form of MreB, as well as with filament formation [50], but MreB polymerization is required for the maintenance of a stable interaction with RodZ [52]. Binding between the two proteins occurs through both a helix-turn­helix motif and a juxtamembrane region of RodZ; this interaction was quantified and mutations that interfere with it affect cell shape and impair RodZ’s ability to localize in a helical fashion along the cell axis [49,50,53]. Interestingly, suppressor mutations of *E. coli rodZ* deletion mutants that restored the rod-like shape could be mapped onto MreB’s subdomain IA [54]. Despite the fact that this does not correspond to the interaction region identified in the crystal structure, it suggests that domains IA and IIA could be proximal in a filamentous form of MreB, explaining the effect of the suppressor mutations [54]. These observations indicate that targeting both the direct RodZ-MreB interaction region (domain IIA) and the surface of domain IA with small molecule inhibitors could prove to be a successful means to disrupt cell shape.

Interactions between MreB and RodZ, PBP2 and RodA have been recently quantified by FRET [52]. This elegant work revealed that FRET signal measured between pairs of molecules (MreB-RodZ; MreB-PBP2; RodA-PBP2) could be disrupted by the addition of the MreB inhibitor A22, or the PBP transpeptidation inhibitor mecillinam. These exciting results not only provided a measurable value for the interaction between PBP2 and RodA for the first time, but also indicated that these interactions are very interesting potential targets for novel small molecule inhibitors of the PG biosynthetic pathway [52]. In addition, BiFC (bimolecular fluorescence complementation) experiments performed using YFP-tagged RodZ also identified interactions with PBPs, RodA, and MreD [53] confirming these specific protein partners as attractive inhibitor development targets.

## 6. During Cell Division: Regulators and Modulators of FtsZ

Another key cytoskeletal element, FtsZ, is the central player in the process of cell division, orchestrating assembly of the divisome through formation of the Z-ring and recruitment of other cell division-related proteins [17,55,56]. The Z-ring is composed of multiple FtsZ protofilaments assembled midcell. During the cell division process, the Z-ring contracts continuously, which explains its dynamic nature and the requirement for its tight regulation [57,58]. The inhibition of FtsZ leads to an arrest in cell division, filamentation, and eventually cell death [6,59,60].

FtsZ is the bacterial homolog of tubulin and folds into a 45 kDa monomer with two key regions: the GTP-binding site and the T7 loop. Polymerization is GTP-dependent, and occurs in a head-to-tail fashion. This is explained by the fact that upon protofilament formation, the T7 loop from one monomer packs in proximity to the nucleotide-binding site of an adjacent molecule, forming a complete GTP-binding cleft [61,62,63,64]. Recently, FtsZ-like proteins identified in archaea have also been linked to cellular shape control and swimming [65].

Regulating proteins such as FtsA, ZipA, SulA, ClpX, ZapC and MciZ intervene at different moments of the contraction process and bind to different sites on FtsZ. Interestingly, bacteria do not all express the same FtsZ-regulating proteins, indicating that distinct regulation mechanisms have been adapted for different cellular requirements (division, sporulation, *etc.*) [17]. Two regulators that have been structurally characterized in complex with FtsZ and that are involved in the first steps of cytokinesis are FtsA and ZipA. At the first stage of ring assembly, FtsA plays an important role in tethering FtsZ to the cytoplasmic membrane. The crystal structure of the FtsA in complex with a peptide from the C-terminus of FtsZ indicates that it is through this region that the Z-ring could be tethered to the membrane [66,67]. In the absence of FtsZ, FtsA also forms filaments that have been reported as either being straight or twisted, attesting to the inherent interdomain flexibility of the molecule [68,69].

The C-terminus of FtsZ has also been demonstrated to bind to ZipA, a membrane-associated protein that localizes to the site of cell division at a very early stage of the division cycle [70]. In the structure of ZipA complexed to a peptide from the C-terminus of FtsZ (Figure 4C), a vast hydrophobic region of ZipA is implicated in binding the helical peptide [71]. A comparison of the two complexed structures (Figure 4B,C) reveals that the C-terminus of FtsZ must adopt different conformations to bind to either of the two proteins, but simultaneous binding is unlikely [66]. Recently, TIRF (total internal reflection fluorescence) experiments showed that ZipA recruits FtsZ monomers to the membrane, whilst FtsA preferentially interacts with polymerized FtsZ, suggesting a more important role for FtsA in cytoskeletal filament formation [72]. It is of note that in Gram-positive and cyanobacteria, the filament-forming protein SepF is involved in FtsZ recruitment to membranes, suggesting an explanation as to why in organisms such as *Bacillus subtilis* FtsA is not required for growth [73].

FtsZ modulators have also attracted interest since they determine cellular responsiveness to environmental stress or nutritional and developmental states. An example is SulA (suppressor of LonA), a “checkpoint” protein that is induced in response to stress and DNA damage in *E. coli*. SulA blocks Z-ring formation by sequestering the FtsZ monomers to which it is bound, reducing the effective concentration of active FtsZ until DNA damage is repaired by the cell [74]. SulA binds directly to the T7 loop of FtsZ, on the opposite side of the GTP-binding pocket (Figure 4D), thus preventing polymerization both by protofilament disassembly and by capping the free end of preformed FtsZ filaments [75]. Upon DNA repair, SulA is proteolyzed [76], which provides a means of regulation of its activity.

Another example is MciZ (for mother cell inhibitor of FtsZ), a 40-amino acid peptide expressed during sporulation in *Bacillus subtilis*. Once expressed, the presence of MciZ in the cytoplasm blocks Z-ring formation by capping the minus (polymerization) end of FtsZ filaments [77,78]. The crystal structure of the complex between FtsZ and MciZ reveals that the latter binds to the C-terminal β-sheet of FtsZ (green in Figure 4E), thus adding two additional strands to the four-stranded region. In this structure, the T7 loop is not traceable, potentially due to flexibility; however, it is hypothesized that MciZ does not prevent polymerization by T7 loop displacement, but through direct steric hindrance. Light-scattering and electron microscopy studies showed that MciZ’s effect of FtsZ polymerization is substoichiometric, and that it binds to the minus end of the polymer, generating shorter filaments [78]. It is of interest that both molecules, SulA and MciZ, bind to FtsZ at its minus end, but block polymerization using distinct mechanisms (SulA: monomer sequestration; MciZ, filament capping) [74,77,78,79]. In addition, SulA displays 10-fold less affinity for FtsZ than MciZ, which may also affect the mechanistic difference [78].

Both direct and indirect evidence has been used to suggest that FtsZ recruits MreB to the Z ring at mid-cell, coupling elements of the divisome to the cell elongation machinery [60]. Immunofluorescence microscopy studies on *E. coli* and *Caulobacer crescentus* indicated that MreB forms a ring-like pattern at mid-cell that co-localizes with Z-rings [80,81], while impairment of MreB function gives both cell elongation and cell division phenotypes [82]. More recently, bacterial and yeast two-hybrid studies indicated a direct FtsZ-MreB interaction, data that was validated by *in vivo* cross-linking. The same authors were able to show that MreB could be recruited to the *E. coli* septum, and a single mutation was able to disrupt the interaction with FtsZ, blocking cell division and recruitment of PBPs to the Z ring [83]. Thus, the MreB-FtsZ interaction mediates the transfer of cell wall synthesis proteins such as PBPs from the elongation to cell division complexes. Other proteins, such as MurG and MraY, have been shown to be essential for both processes, and it remains to be determined if they also require recruitment by MreB [83].

In addition to modulators and regulating proteins, the FtsZ filament can also be influenced by the action of other cytoskeletal elements. MinC and MinD form a copolymer that prevents the Z-ring from assembly anywhere in the cell but in the septal region, thus preventing aberrant cell division [84]. Recently, it was shown that MinC and MinD form an alternating copolymer that can bind directly to FtsZ filaments as well as to the bilayer. The nature of the MinCD filament is of particular interest, since it is formed by two structurally distinct proteins (Figure 4F) with the MinD interacting sites, characterized by mutagenesis, being located on the opposite sides of MinC [85,86]. Authors suggest that the MinCD copolymer regulates FtsZ filament formation by recruiting the polymer itself, and not monomeric FtsZ, to the membrane. Bridging would be accomplished through the C-terminus of MinC, which would then keep the FtsZ ring at a certain distance from the bilayer (approx. 16 nm). These observations thus suggest that MinCD regulation of FtsZ ring formation does not involve disruption of filament formation, as previously hypothesized, but rather acts through a preferential interaction with the FtsZ polymer [85]. Despite the fact that these exciting data suggest that targeting the MinC-FtsZ interaction interface could be a novel strategy for inhibitor development, the role of MinCD copolymers in FtsZ anchoring has recently been questioned [87], necessitating further investigation of the role of this potential interaction in bacterial cell division.

## 7. Concluding Remarks

The sheer complexity of the bacterial cell wall biosynthetic pathway has created significant challenges for the characterization of proteins that are involved in the different aspects of the process. One main difficulty for the structural study of complexes of proteins involved in any step of peptidoglycan biosynthesis has been the fleeting nature of many of the interactions; in a recent study, *E. coli* divisome components were reported to form a 1 megadalton complex in rapidly dividing cells, but dissociated once these cells had reached the stationary growth phase [56]. The same authors cautioned that protein purification techniques that are classically employed in laboratories, such as French press or sonication followed by centrifugation, often disrupt the divisome, which was described as a “loose assembly of proteins.” These observations attest to the difficulty of the objective at hand in terms of structural biology of protein complexes: to obtain stable, homogeneous samples that are amenable to structural techniques. Despite the challenging objectives, the results summarized here are a reflection of the fact that methodologies that can be used to attain them are now starting to become available, and the near future should allow many more details of protein interactions involved in these complex machineries to come into view.
